# SGLT-2 inhibitors on prognosis and health-related quality of life in patients with heart failure and preserved ejection fraction: A systematic review and meta-analysis

**DOI:** 10.3389/fcvm.2022.942125

**Published:** 2022-09-08

**Authors:** Danning Yang, Yu Zhang, Jie Yan, Ming Liu, Fengshuang An

**Affiliations:** Key Laboratory of Cardiovascular Remodeling and Function Research, Chinese Ministry of Education, Chinese National Health Commission and Chinese Academy of Medical Sciences, State and Shandong Province Joint Key Laboratory of Translational Cardiovascular Medicine, Department of Cardiology, Qilu Hospital, Cheeloo College of Medicine, Shandong University, Jinan, China

**Keywords:** sodium-glucose cotransporter-2 inhibitors, heart failure with preserved ejection fraction, prognosis, health-related quality of life, meta-analysis

## Abstract

**Background:**

Heart failure with preserved ejection fraction (HFpEF) is becoming the main subtype of heart failure, but lacks proven effective therapies. Sodium-glucose cotransporter-2 (SGLT-2) inhibitor, a new kind of oral glucose-lowering agent, shows a great effect on improving cardiovascular outcomes. Based on the results of current RCTs, we perform this meta-analysis to illustrate the therapeutic impact of SGLT2i in HFpEF patients.

**Methods:**

We systematically searched the online database and 10 RCTs were involved. The primary outcome was the prognosis outcome of HFpEF patients, including a composite outcome of cardiovascular (CV) death and hospitalization for heart failure (HHF), CV mortality, HHF, and all-cause mortality. Main secondary outcomes included improvement of KCCQ-TSS (Kansas City Cardiomyopathy Questionnaire and total symptom score) and 6-Minute Walk Test (6MWT). All pooled results were calculated by the random-effects model. Statistical heterogeneity was assessed using the chi-squared test and was quantified using the I-squared statistic.

**Results:**

Ten RCTs comprising 10,334 patients were involved in. Incidence of composite outcome was reduced in SGLT-2 inhibitor group compared with placebo (HR: 0.78, 95% CI: 0.69–0.88, *p* = 0.00). Improvement of KCCQ-TSS was also more pronounced in the SGLT-2 inhibitor group (MD: 2.74, 95% CI: 1.30–4.18, *p* = 0.00). No statistical difference was observed in 6MWT.

**Conclusion:**

Treating HFpEF patients with SGLT-2 inhibitors is associated with reducing the composite outcome of CV death and HHF and improving health-related quality of life. Further studies with more evidence are in need to confirm this conclusion.

## Introduction

Heart failure with preserved ejection fraction (HFpEF), previously known as diastolic heart failure, is a subtype of heart failure characterized as left ventricular ejection fraction (LVEF) ≥50%. Until now, HFpEF has become the predominant form of heart failure worldwide, especially in aged population (1, 2). Despite recognizing the complexity of the specific clinical syndrome in the recent 20 years, the pathophysiology of HFpEF has not been illustrated explicitly. In consideration of the unclear mechanism, nor do people find a treatment that has a convincing clinical benefit in reducing mortality or morbidity of HFpEF. Compared with the recommended quadruple regimen of HFrEF, no Ia or Ib class recommendations were released in the 2021 ESC guideline (3).

Sodium-glucose cotransporter-2 (SGLT-2) inhibitor is a new class of oral glucose-lowering agents that can promote glucosuria and thus reduce serum glucose and lower blood pressure. A large amount of evidence has emerged that other than increasing glucosuria excretion in T2DM patients, SGLT-2 inhibitors can significantly improve cardiovascular outcomes, including decreasing the incidence of cardiovascular (CV) death and hospitalization for heart failure (HHF) (4–6). In the field of heart failure, meta-analysis of several large-scale RCTs has convinced that SGLT-2 inhibitors can reduce all-cause and cardiovascular death in HFrEF patients (7, 8).

As a result of the potential benefits, large-scale RCTs were also implemented to determine the effect SGLT-2 inhibitors might have on HFpEF. EMPEROR-Preserved trial, released in 2021, showed that empagliflozin reduced the combined cardiovascular risk in HFpEF patients, which made it the first large RCT to achieve a positive endpoint (9). Therefore, collecting existing RCTs, we performed this systematic review and meta-analysis to evaluate the prognosis of HFpEF population treated with SGLT-2 inhibitors. To understand health-related quality of life (HRQoL) and exercise capacity in the population, we also evaluated the improvement of the Kansas City Cardiomyopathy Questionnaire (KCCQ) score,6-Minute Walk Test (6MWT), and NT-proBNP levels.

## Methods

This systematic review and meta-analysis followed the recommendations of the Preferred Reporting Items for Systematic Reviews and Meta-analyses (PRISMA) statement (10) and the Cochrane Handbook for Systematic Reviews of Interventions (11). The entire process of our meta-analysis abides by PICOS criteria.

### Data sources and search methods

Two independent investigators (DN.Y. and Y.Z.) independently searched the following online databases: PubMed, Embase, Cochrane Library, ClinicalTrials.gov, and SinoMed from the establishment of the databases till 5 May 2022. Search terms included MeSH terms “Heart Failure”, “Heart Failure, Diastolic”, “Sodium-Glucose Transporter 2 Inhibitors” and all relevant entry terms. A detailed searching strategy is shown in Supplementary materials. To ensure no relevant publications were overlooked, we also manually searched for qualifying publications in the reference lists of eligible articles. Only randomized controlled trials could be involved. No date limit was put in this meta-analysis.

### Endpoints and study selection

The primary outcome of this meta-analysis was the prognosis outcome of HFpEF patients, including a composite outcome of CV death and HHF, CV mortality, HHF, and all-cause mortality. The main secondary outcome was the improvement of KCCQ-TSS (Kansas City Cardiomyopathy Questionnaire, Total Symptom Score) and 6MWT. Other secondary outcomes included improvement of KCCQ-CSS (Clinical Summary Score), KCCQ-OSS (Overall Summary Score), KCCQ-PL (Physical Limitation), and NT-proBNP level.

The studies included in our meta-analysis must meet all of the following criteria: (a) randomized, double-blind, placebo-controlled trials; (b) involve the HFpEF population, defined as patients with symptoms and signs of HF, with evidence of structural and/or functional cardiac abnormalities and/or raised natriuretic peptides (NPs), and with an LVEF > 40%; (c) compare SGLT-2 inhibitors with another placebo, or add SGLT-2 inhibitors in a standard diabetic therapy; (d) report any of the following outcomes: a composite outcome of CV death and HHF, all-cause mortality, KCCQ score of subscales, 6MWT, and NT-proBNP level. Publications were excluded from the meta-analysis if they were (a) conference reports, reviews, case reports, or summaries; (b) studies published in a language other than English or Chinese; and (c) head-to-head studies compared SGLT-2 inhibitors with other glucose-lowering agents.

### Data collection and quality assessment

Two reviewers (DN.Y. and Y.Zh.) independently extracted data of interest using an electronic data collection form designed preciously. The extracted data mainly include the following: (a) Baseline characteristics of included studies: trial name and trial number, study design, subgroup population, details of treatments, publication time, and follow-up duration; (b) Baseline characteristics of patients: gender, age, NYHA class, LVEF, NT-proBNP level, blood pressure, eGFR, and so on; (c) Study outcomes: A composite outcome of CV death and HHF, CV mortality, HHF, all-cause mortality, KCCQ score of subscales, 6MWT, and NT-proBNP level.

The quality of each study was assessed by two reviewers (DN.Y. and Y.Zh.) separately using the Cochrane Collaboration's tool for assessing risk of bias, which includes the following seven domains: random sequence generation (selection bias), allocation concealment (selection bias), blinding of participants and personnel (performance bias), blinding of outcome assessment (detection bias), incomplete outcome data (attrition bias), selective reporting (reporting bias), and other bias (12). All disagreements were resolved with consensus by a third reviewer (J.Y.)

### Statistical analysis and certainty of the evidence

This meta-analysis was performed using Stata16.0. For the composite outcome of CV death and HHF, we extracted hazard ratios (HRs) and corresponding 95% confidence intervals (95% CIs) to make a pooled estimate. For CV mortality, HHF, and all-cause mortality, we used odd ratios (ORs) to measure the intervention effects of dichotomous outcomes. For KCCQ and 6MWT, we extracted adjusted mean differences (MDs) and 95% CIs from original studies. The level of NT-proBNP was analyzed using SMD. The random-effects model was used to calculate the pooled effect, and only when *p* < 0.05, the results were considered to be statistically meaningful. Statistical heterogeneity was assessed using the chi-squared test (*p* < 0.10 was considered statistically significant for heterogeneity) and was quantified using the *I*-squared statistic (*I*^2^ > 50% was considered substantial heterogeneity). Sensitivity analysis was performed to examine the robustness of the results and the effect of potential effect modifiers.

Evidence summaries were prepared for each outcome using GRADEprofiler (Version 3.6). Certainty of the evidence was assessed using the GRADE (Grading of Recommendations Assessment, Development, and Evaluation) system. The confidence of the estimate effect can be categorized into four levels: very low, low, moderate, and high, and RCTs are classified into the highest grade. Reasons for downgrading the evidence included risk of bias, inconsistency, indirectness, imprecision, and publication bias (13).

## Results

**Figure 1:**
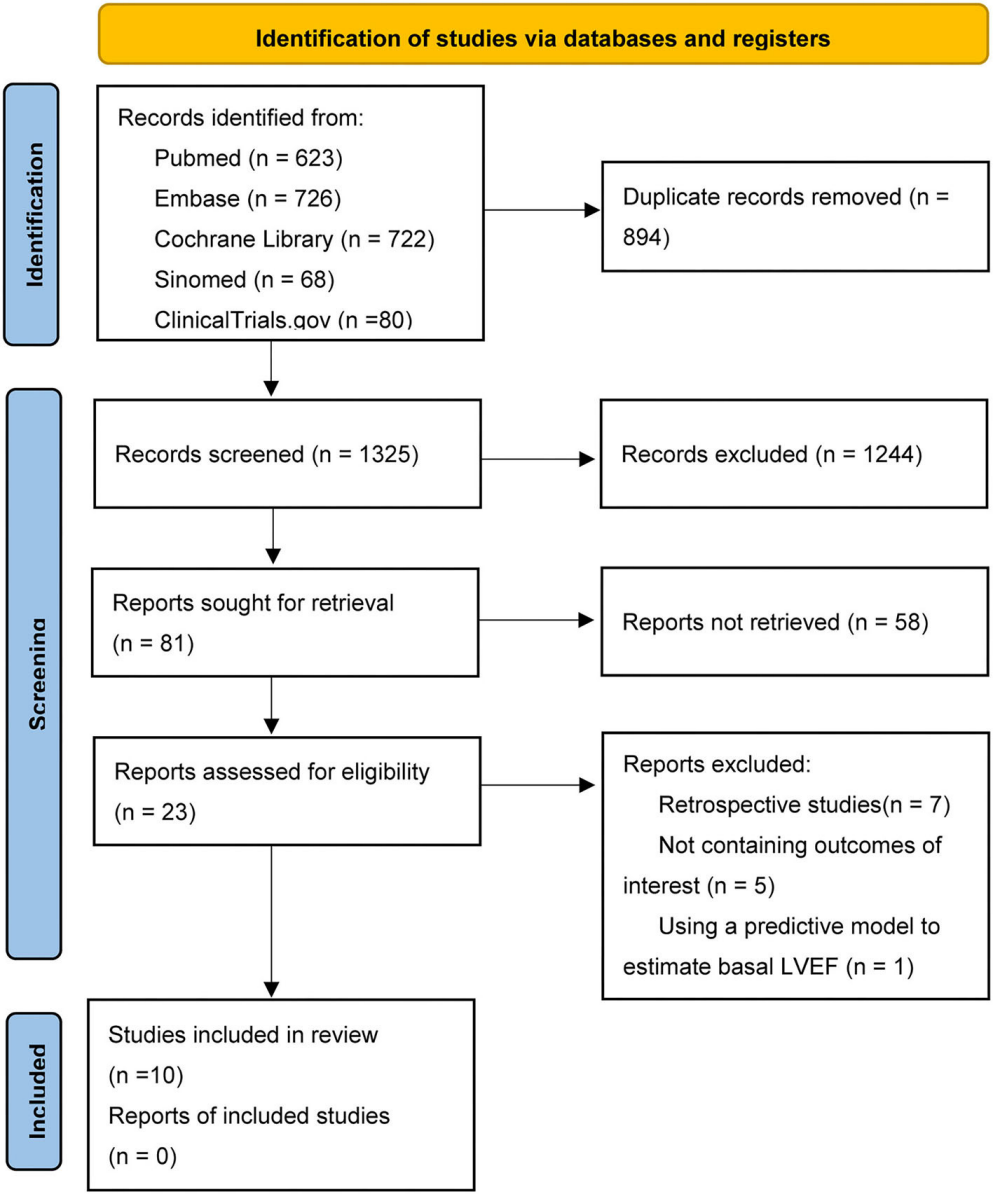
PRISMA flowchart of selection.

The selection process is shown in Figure 1. We searched a total of 2,219 literature, and among them, 1,325 studies were screened based on the abstracts and titles. Finally, 11 studies involving 10 RCTs were included in this meta-analysis (9, 14–23). It should be mentioned that among them, the DETERMINE-preserved trial has not been published yet, whereas we included the results published in ClinicalTrials.gov (23). About 10,334 patients who met our inclusion criteria were randomized to either the SGLT-2 inhibitor group or placebo group. There were four dapagliflozin trials (14, 20, 22, 23) (1,636 patients), two empagliflozin trials (9, 16, 17) (6,203 participants), two sotagliflozin trials (18, 19) (921 participants), one ertugliflozin trial (15) (1,007 participants), and one canagliflozin trial (21) (267 participants). The characteristics of included studies and patients are shown in Table 1.

**Table 1A:** Basal characteristics of included studies.

**Clinical trial**	**Publication time**	**Study design**	**Intervention**	**Follow-up duration**	**LVEF inclusion criteria**	**T2DM,%**	**Total amount**	End points^*^
DECLARE-TIMI 58 NCT01730534	2019	Randomized Controlled Trial	Dapagliflozin/10 mg qd placebo	4.2 years	LVEF ≥ 45%	100%	808	1,2,3,4
VERTIS CV NCT01986881	2020	Randomized Controlled Trial	Ertugliflozin/5 mg qd or 15 mg qd placebo	3.5 years	LVEF > 45%	100%	1,007	1,2,3,4
EMPERIAL-Preserved NCT03448406	2020	Randomized Controlled Trial	Empagliflflozin/10 mg qd placebo	12 weeks	LVEF > 40%	51.10%	315	4,5,6,7
EMPEROR-Preserved NCT03057951	2021	Randomized Controlled Trial	Empagliflozin/10 mg qd placebo	26.2 months	LVEF > 40%	49.10%	5,988	1,2,3,4,5
SOLOIST-WHF NCT03521934	2021	Randomized Controlled Trial	Sotagliflozin/200 mg qd placebo	9 months	LVEF ≥ 50%	100%	256	1
SCORED NCT03315143	2021	Randomized Controlled Trial	Sotagliflozin/200 mg qd placebo	16 months	LVEF ≥ 50%	100%	665	1
PRESERVED-HF NCT03030235	2021	Randomized Controlled Trial	Dapagliflozin/10 mg qd placebo	12 weeks	LVEF ≥ 45%	55.90%	324	4,5,6,7
TANG Xiaodi, FAN Ying, etc.	2021	Randomized Controlled Trial	Dapagliflozin/10 mg qd conventional treatment	24 weeks	LVEF ≥ 50%	100%	200	4,7
CHIEF-HF NCT04252287	2022	Randomized Controlled Trial	Canagliflozin/100 mg qd placebo	12 weeks	LVEF ≥ 45%	Not 100%, NA	267	5
DETERMINE-preserved NCT03877224	No publication	Randomized Controlled Trial	Dapagliflozin/10 mg qd placebo	16 weeks	LVEF > 40%	Not 100%, NA	504	4,5,6

LVEF, left ventricular ejection fraction; T2DM, type 2 diabetes mellitus.

* Endpoints: 1. CV death and HHF; 2. hospitalization for heart failure; 3. cardiovascular mortality; 4. all-cause mortality; 5. KCCQ; 6. 6MWT; 7. NT-proBNP level.

**Table 1B:** Basal characteristics of included studies.

**Clinical trial**	**Intervention**	**Patient size**	**Age, y**	**Male, %**	**Body mass index, kg/m^2^**	**LVEF, %**	**NYHA**				**Heart rate, bpm**	**Systolic pressure, mmHg**	**Median NT-proBNP concentration, pg/mL**
							**I**	**II**	**III**	**IV**			
DECLARE-TIMI 58	Dapagliflozin	399	NA	NA	NA	NA	NA	NA	NA	NA	NA	NA	NA
	Placebo	409	NA	NA	NA	NA	NA	NA	NA	NA	NA	NA	NA
VERTIS CV	Ertugliflozin	680	63.8 ± 8.3	65.6	32.6 ± 5.3	NA	22.5	67.1	7.1	0.1	NA	NA	NA
	Placebo	327	64.7 ± 8.2	63.3	32.9 ± 5.3	NA	25.7	67.6	4.6	0	NA	NA	NA
EMPERIAL-Preserved	Empagliflflozin	157	73.6 ± 8.2	55.4	30.3 ± 5.8	51.9 ± 9.7	0.7	74.5	24.8	0	70.6 ± 12.7	127.6 ± 18.7	966 (572, 1,653)
	Placebo	158	74.6 ± 9.7	58.2	29.3 ± 5.0	52.6 ± 9.7	0	79.7	20.3	0	70.4 ± 12.7	132.1 ± 18.7	843 (407, 1,913)
EMPEROR-Preserved	Empagliflozin	2997	71.8 ± 9.3	55.4	29.8 ± 5.8	54.3 ± 8.8	0.1	81.1	18.4	0.3	70.4 ± 12.0	131.8 ± 15.6	994 (501, 1,740)
	Placebo	2991	71.9 ± 9.6	55.3	29.9 ± 5.9	54.3 ± 8.8	<0.1	81.9	17.8	0.3	70.3 ± 11.8	131.9 ± 15.7	946 (498, 1,725)
SOLOIST-WHF	Sotagliflozin	127	NA	NA	NA	NA	NA	NA	NA	NA	NA	NA	NA
	Placebo	129	NA	NA	NA	NA	NA	NA	NA	NA	NA	NA	NA
SCORED	Sotagliflozin	NA	NA	NA	NA	NA	NA	NA	NA	NA	NA	NA	NA
	Placebo	NA	NA	NA	NA	NA	NA	NA	NA	NA	NA	NA	NA
PRESERVED-HF	Dapagliflozin	162	70.1 ± 9.7	43.2	35.8 ± 8.5	60.0 ± 7.5	59.3	40.1		0.6	69.3 ± 12.0	135.4 ± 23.9	641 (373, 1,210)
	Placebo	162	70.6 ± 11.2	43.2	34.9 ± 8.0	59.6 ± 8.2	55.6	44.4		0	68.4 ± 9.7	132.7 ± 22.4	710 (329, 1,449)
TANG Xiaodi,etc.	Dapagliflozin	100	63.4 ± 11.0	61	25.0 ± 3.2	NA	0	62.0	38.0	0	NA	NA	938 (469, 1,407)
	Conventional treatment	100	63.6 ± 14.8	60	25.4 ± 3.1	NA	0	69.0	31.0	0	NA	NA	932 (466, 1,398)
CHIEF-HF	Canagliflozin	132	NA	NA	NA	NA	NA	NA	NA	NA	NA	NA	NA
	placebo	135	NA	NA	NA	NA	NA	NA	NA	NA	NA	NA	NA
DETERMINE-preserved	Dapagliflozin	253	72.0 ± 9.1	64	NA	NA	NA	NA	NA	NA	NA	NA	NA
	Placebo	251	71.7 ± 9.7	62	NA	NA	NA	NA	NA	NA	NA	NA	NA

Data reported as mean ± SD or median (interquartile range).

LVEF, left ventricular ejection fraction; NYHA, New York Heart Association; bpm, beats/minute.

**Figure 2:**
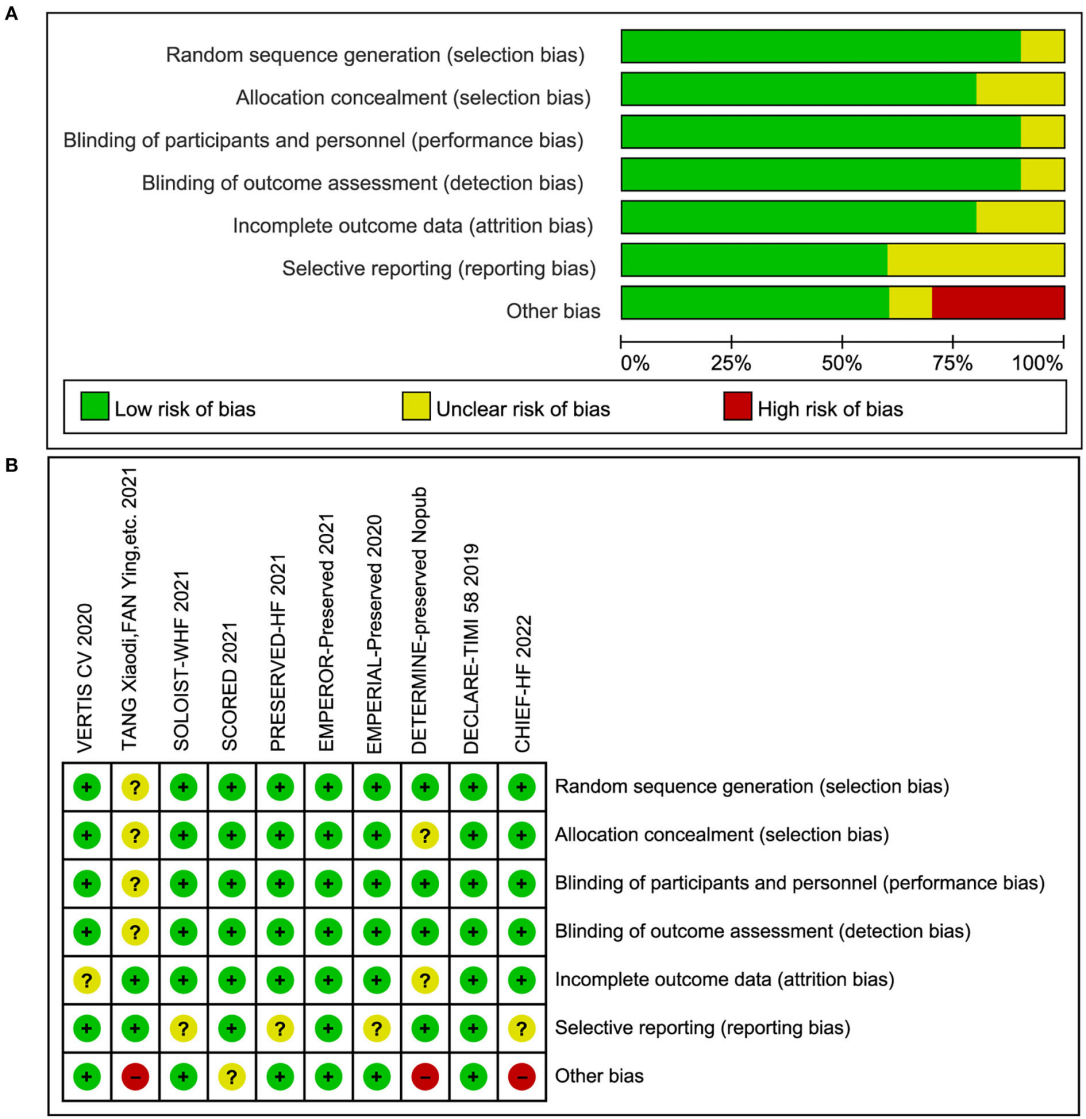
**(A)** Risk of bias graph. **(B)** Risk of bias summary.

Quality assessment is shown in Figure 2. Three of the 10 studies had a relatively greater risk of bias because of the possible mistakes during the trial. Given that no more than 10 studies were involved in each outcome, no funnel plot was conducted to detect publication bias.

### Cardiovascular mortality and hospitalization for heart failure

**Figure 3:**
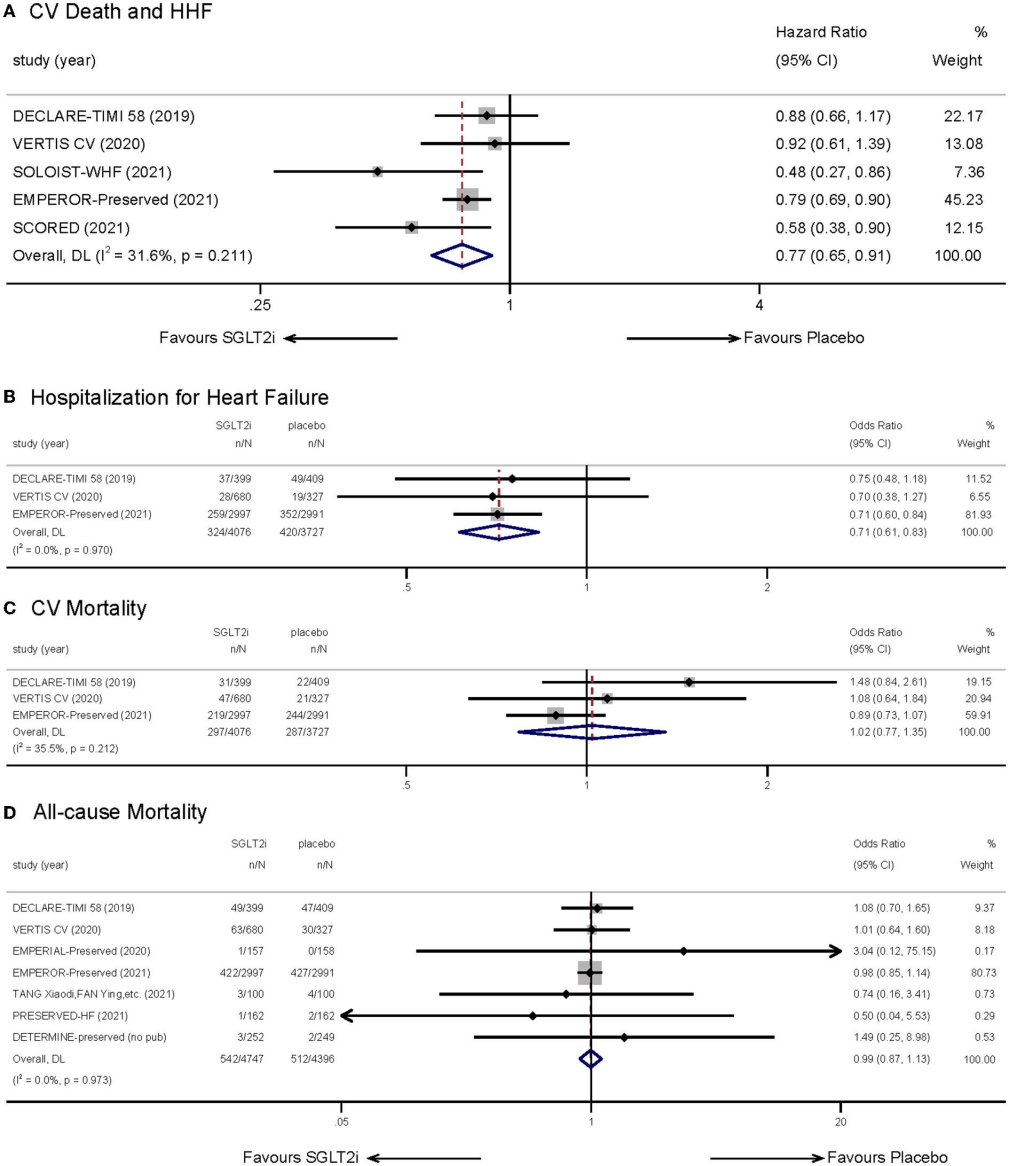
Effect of SGLT-2 inhibitors vs. placebo on the primary outcome of **(A)** cardiovascular death and hospitalization for heart failure; **(B)** hospitalization for heart failure; **(C)** cardiovascular mortality; **(D)** all-cause mortality.

Five studies (9, 14, 15, 18, 19), accessible for their hazard ratios (HRs) and corresponding 95% confidence intervals (CIs), were selected to evaluate the effects of SGLT-2 inhibitors on a composite of CV death and HHF. Meta-analysis showed that treating with SGLT-2 inhibitors decreased the incidence of the composite outcome (HR: 0.77, 95% CI: 0.65–0.91, *p* = 0.00; Figure 3A). No obvious statistical heterogeneity was observed between the studies (*I*^2^ = 31.6%, *p* = 0.211). When eliminating each study, the result kept stable. Subgroup analysis illustrated a consistency no matter whether patients had T2DM (Supplementary Figure 1).

Combination of three relevant trials (9, 14, 15) indicated that treatment with SGLT-2 inhibitors could lower incidence of hospitalization for heart failure (OR: 0.71, 95% CI: 0.61–0.83, *p* = 0.00; *I*^2^ = 0.00%, *p* = 0.970; Figure 3B). However, we did not observe significant difference in CV mortality (9, 14, 15) (OR: 1.02, 95% CI: 0.77–1.35, *p* = 0.888; *I*^2^ = 35.5%, *p* = 0.212; Figure 3C) when treating with SGLT2i. Seven studies (9, 14–16, 20, 22, 23) reported statistics to do with all-cause mortality. The results indicated that SGLT-2 inhibitors showed no advantage in reducing all-cause mortality (OR: 0.99, 95% CI: 0.87–1.13, *p* = 0.936; *I*^2^ = 0.00%, *p* = 0.973; Figure 3D). In consideration that the follow-up duration of included studies showed a great difference, we implemented a subgroup analysis which showed no difference (Supplementary Figure 2).

### Health-related quality of life

**Figure 4:**
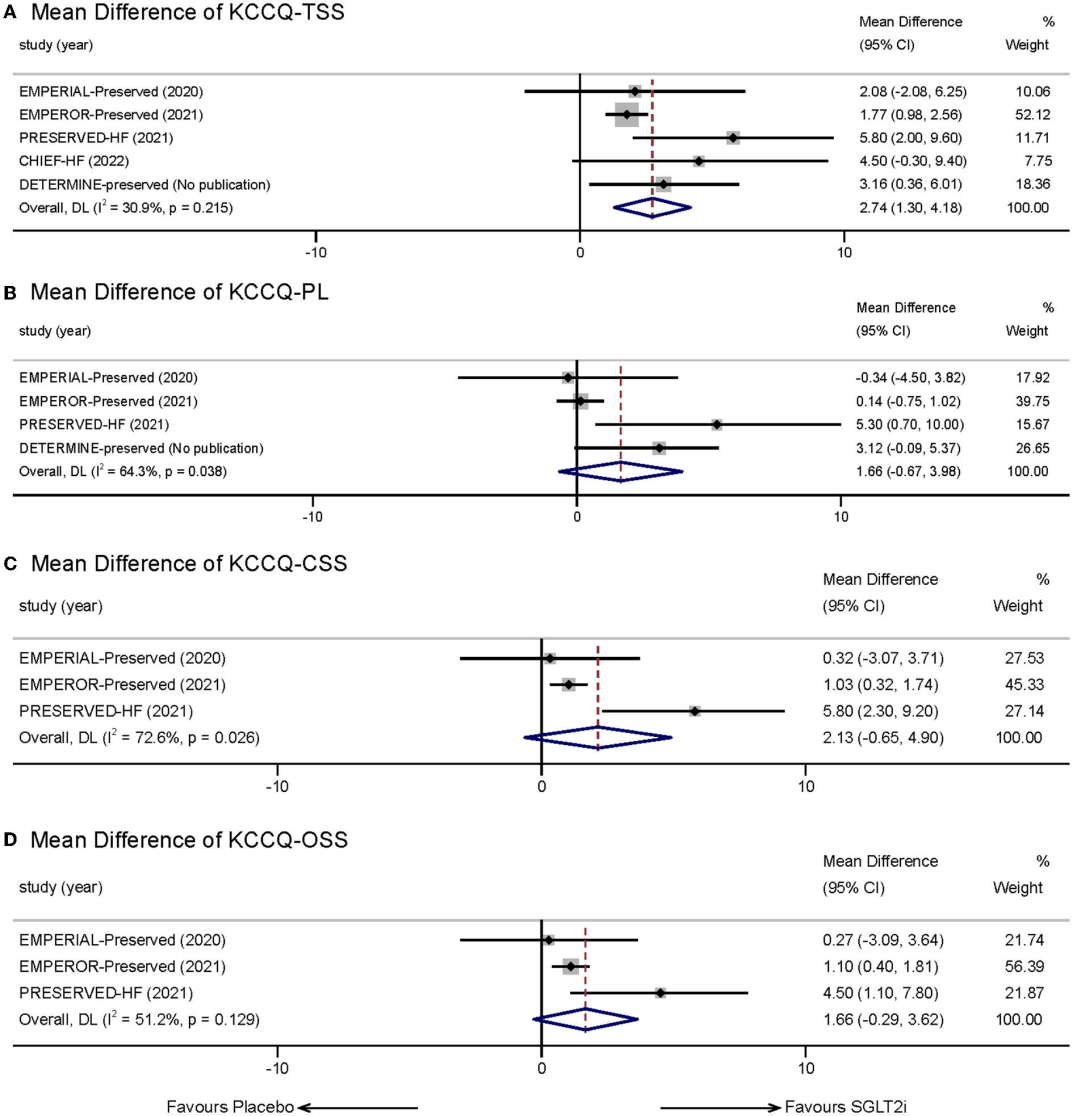
Effect of SGLT-2 inhibitors vs. placebo on KCCQ Subscales: **(A)** KCCQ-TSS; **(B)** KCCQ-PL; **(C)** KCCQ-CSS; **(D)** KCCQ-OSS.

Five studies (9, 16, 17, 20, 21, 23) reported the therapeutic effect of SGLT-2 inhibitors in HFpEF patients using health-related quality of life outcomes measured by the KCCQ-23 Scale. Given that we set KCCQ-TSS as our main observation subscale, after estimating, the SGLT2i group showed a greater improvement in KCCQ-TSS from baseline compared with placebo (MD:2.74, 95% CI: 1.30–4.18, *p* = 0.00; Figure 4A). Statistical heterogeneity between the studies was not present (*I*^2^ = 30.9%, *p* = 0.215). The result remained stabilized when we eliminated each study one by one.

We also measured other subscales of KCCQ reported in the incorporated trials, including KCCQ-PL, KCCQ-CSS, and KCCQ-OSS. Four studies reported statistics of KCCQ-PL. The mean treatment difference between the two groups was not significant (MD:1.66, 95% CI: −0.67 to 3.98, *p* = 0.162; *I*^2^ = 64.3%, *p* = 0.038; Figure 4B). Three studies reported KCCQ-CSS and KCCQ-OSS. However, meta-analysis also indicated that SGLT-2 inhibitors did not show significant effects in the two aspects compared with placebo (KCCQ-CSS: MD: 2.13, 95% CI: −0.65 to 4.90, *p* = 0.133; *I*^2^ = 72.6%, *p* = 0.026; Figure 4C; KCCQ-OSS: MD:1.66, 95% CI: −0.29 to 3.62, *p* = 0.096; *I*^2^ = 51.2%, *p* = 0.129; Figure 4D).

### Exercise capacity outcomes

**Figure 5:**
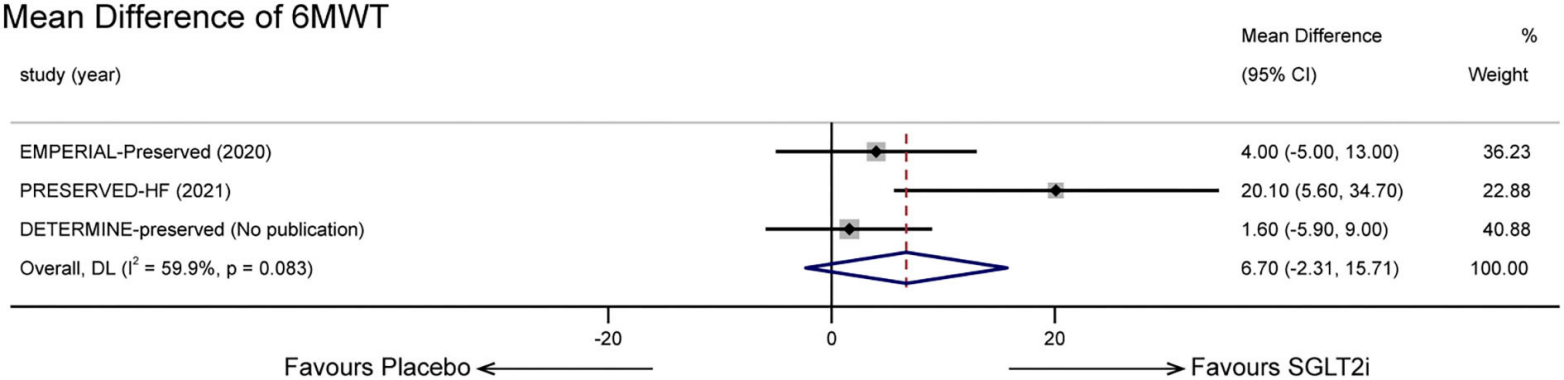
Effect of SGLT-2 inhibitors vs. placebo on 6-Minute Walking Test.

Three trials (16, 20, 23) worked on 6MWT. However, our research did not indicate that short-term treatment with SGLT-2 inhibitors would improve exercise capacity (MD: 6.70, 95% CI: −2.31 to 15.71, *p* = 0.145; Figure 5). Statistical heterogeneity between the studies was present (*p* = 0.083, *I*^2^ = 59.9%). When we excluded the PRESERVED-HF trial, the overall MD was 2.58 m (95% CI −3.16 to 8.31, *p* = 0.379) and there was no significant heterogeneity (*p* = 0.687, *I*^2^ = 0%).

### Serum NT-proBNP level

**Figure 6:**
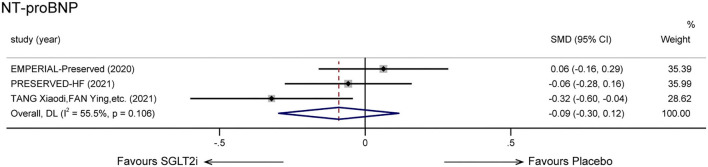
Effect of SGLT-2 inhibitors vs. placebo on NT-proBNP.

Three trials (16, 20, 22) provided accessible statistics associated with serum NT-proBNP levels. We could not observe a significantly statistical difference in SGLT2i therapy group compared with other treatments (SMD: −0.09, 95% CI: −0.30 to 0.12, *p* = 0.388; *I*^2^ = 55.5%, *p* = 0.106; Figure 6).

### Certainty of evidence

Certainty of evidence, as shown in Table 2, was evaluated by GRADE methodology. All studies included were RCTs and thus were originally classified into the highest grade. After evaluation of primary and main secondary outcomes, exercise capacity was degraded to moderate evidence as a result of the presentation of heterogeneity.

**Table 2:** Summary of findings.

**Outcome**	**Quality assessment**							**Effect**		**Quality**
	**No of studies**	**Design**	**Risk of bias**	**Inconsistency**	**Indirectness**	**Imprecision**	**Other considerations**	**Relative (95% CI)**	**Absolute**	
CV Death and HHF	5	randomised trials	no serious risk of bias	no serious inconsistency	no serious indirectness	no serious imprecision	none	HR 0.78 (0.69 to 0.88)	22 fewer per 1000 (from 12 fewer to 31 fewer)	⊕⊕⊕⊕ HIGH^2^
HHF	3	randomised trials	no serious risk of bias	no serious inconsistency	no serious indirectness	no serious imprecision	none	OR 0.71 (0.61 to 0.83)	30 fewer per 1000 (from 17 fewer to 41 fewer)	⊕⊕⊕⊕ HIGH^2^
CV Mortality	3	randomised trials	no serious risk of bias	no serious inconsistency	no serious indirectness	no serious imprecision	none	RR 1.02 (0.77 to 1.35)	2 more per 1000 (from 18 fewer to 27 more)	⊕⊕⊕⊕ HIGH^2^
All-cause Mortality	7	randomised trials	no serious risk of bias	no serious inconsistency	no serious indirectness	no serious imprecision	none	OR 0.99 (0.87 to 1.13)	1 fewer per 1000 (from 14 fewer to 13 more)	⊕⊕⊕⊕ HIGH^2^
KCCQ-TSS	5	randomised trials	no serious risk of bias	no serious inconsistency	no serious indirectness	no serious imprecision	none	MD 2.74(1.30 to 4.18)	MD 2.74 higher (1.3 to 4.18 higher)	⊕⊕⊕⊕ HIGH^2^
6-Minute Walking Test	3	randomised trials	no serious risk of bias	serious^1^	no serious indirectness	no serious imprecision	none	MD 6.70(-2.31 to 15.71)	MD 6.70 higher (2.01 lower to 15.71 higher)	⊕⊕⊕ MODERATE^2^

CV, Cardiovascular; HHF, Hospitalization for heart failure; KCCQ, the Kansas City Cardiomyopathy Questionnaire; TSS, Total symptom score; HR, Hazard ratio; OR, Odd ratio; MD, Mean Difference.

1 Statistical heterogeneity showed P < 0.1, I^2^>50%.

2 High quality means further research is very unlikely to change our confidence in the estimate of effect, and moderate quality means further research is likely to have an important impact on our confidence in the estimate of effect and may change the estimate.

## Discussion

This meta-analysis, covering over 10,300 HFpEF patients in 10 prospective studies, explicitly demonstrated that treatment with SGLT-2 inhibitors could lower the incidence of a composite outcome including CV death and HHF in the HFpEF population. In addition, SGLT-2 inhibitors showed a beneficial impact on decreasing events for HHF separately. We also made an analysis to illustrate the amelioration in health-related quality of life after SGLT2i therapy, which specified significant excellence in improving symptom-relevant KCCQ score.

For a long time, it was discouraged when referred to therapeutic medications dealing with HFpEF. It is now widely believed that originated from various risk factors such as overweight, hypertension, and diabetes mellitus (24), complicated physiology and molecular processes are involved in the onset and development of HFpEF, including systematic inflammation, LV structural remodeling, and abnormal hemodynamics (1, 25). Large-scale trials with several medications, including RAAS inhibitors, aldosterone antagonists (MRAs), and angiotensin receptor/neprilysin inhibitors (ARNIs), which had confirmed notable benefits to improve the prognosis of HFrEF patients, did not show superiority for their primary efficacy endpoints in HFpEF (26–28). Despite there existed studies showing merits in some aspects of HFpEF treatment, for example, lowering NT-proBNP levels (29, 30), and improving LV diastolic function (31), these data had not been widely endorsed by international guidelines (32). However, the situation seemed to start changing when the EMPEROR-Preserved group announced their results in 2021. A 21% lower relative risk on the primary outcome compared with placebo group made empagliflozin the first and only drug therapy significantly improving prognosis outcomes of HFpEF, no matter whether T2DM existed (9). In a *post-hoc* analysis of the EMPA-REG OUTCOME trial, empagliflozin similarly reduced the risk of HHF or CV mortality in predicted HFpEF patients with T2DM (33). Compared with some earlier subgroup analyses that proved ineffective in improving prognosis, the efficacy of SGLT-2 inhibitors seemed to be conflicted and needed further exploration. This meta-analysis, making a pooled estimate of several clinical trials related to HFpEF, indicated that (a) intervening with SGLT-2 inhibitors could decrease 23% relative risk of the composite outcome including cardiovascular death and hospitalization for heart failure; (b) the results of primary outcome remained consistent no matter T2DM existed; and (c) the incidence of hospitalization for heart failure was also reduced, whereas CV mortality and all-cause mortality remained unchanged. The strength of SGLT-2 inhibitors in improving prognosis outcomes in HFpEF patients was confirmed in our study, and this could provide direct evidence for the following therapeutic options.

Health-related quality of life (HRQoL) can also be improved in HFpEF patients after treatment with SGLT-2 inhibitors. Patient-reported HRQoL is considered standardized information reflecting a patient's current health status and prognostic implications. Poor HRQoL not only means aggravation of heart failure symptoms and emergency of adverse events but also gives an implication of all-cause death and the composite of death or HF hospitalization, especially in HF patients with a preserved fraction (34, 35). Thus, we evaluated the improvement of HRQoL by making a pooled estimate of KCCQ-23, which has been qualified by the U.S. Food and Drug Administration as a patient-reported clinical assessment tool in heart failure (36). KCCQ Scale, comprising seven domains and 23 items, gives different manifestations of heart failure, including symptoms, functional limitation, and quality of life, by dividing different domains into four subscales—KCCQ-TSS, KCCQ-PL, KCCQ-CSS, and KCCQ-OSS (36). Our meta-analysis put KCCQ-TSS as a main observe indicator as we were more interested in patient-reported improvement in symptoms. In this meta-analysis, treating with SGLT-2 inhibitors could lead to significant 2.74 points higher KCCQ-TSS than the placebo group in total and that means patients are more likely to get remission for heart failure symptoms, such as fatigue, dyspnea, and edema after a same course of treatment. Given that the follow-up duration of the involved studies is relatively short (12 weeks in four trials and 16 weeks in one trial), we can probably infer that short-term treatment with SGLT-2 inhibitors can still improve health status. Another publication of the EMPEROR-Preserved trial revealed Weeks 32 and 52 results of KCCQ score, and a significant increase was observed across all domains of the KCCQ Scale except physical limitation (17). Earlier studies of other medications showed limited evidence in promoting health-related quality of life of HFpEF patients; therefore, our findings of SGLT2i demonstrated the ability on remitting patient-reported HF symptoms. KCCQ-CSS and KCCQ-OSS, with relatively greater heterogeneity, did not show significant differences in our study. The lack of relevant literature and statistics may account for the results. Ongoing clinical trials may provide us with more evidence to clarify the improvement of KCCQ Scales in the coming days.

As for exercise capacity outcomes, no statistically meaningful results were observed. It is generally believed that performance in 6MWT is associated with the health status and prognosis of HF patients (37). Several explanations may explicate the controversial result. First, studies related to 6MWT were limited. The involved patient size was small, and the follow-up duration was relatively short. Second, the heterogeneity between groups was great, and the initial NYHA class was unbalanced. More than 55% of patients in PRESERVED-HF were initially divided into class I, which could explain the greater improvement in the PRESERVED-HF trial. Third, there may be a dissociation between exercise capacity and heart failure, as it can also be influenced by respiratory and musculoskeletal systems (38). Assessing peak oxygen consumption (peak VO_2_) during cardiopulmonary exercise testing (CPET), recommended in recent years, provides more precise information on functional capacity from a cardiovascular intervention compared with 6MWT (39). In the field of HFrEF, there have been studies assessing the effective parameter (40). Regretfully, clinical trials relevant to HFpEF and SGLT2i have yet involved the parameter, and it may be another direction to clarify the functional capacity effect. We did not observe a significant disparity in NT-proBNP levels compared to SGLT-2 inhibitors with placebo as well. In the EMPEROR-Preserved trial, mean differences emerged after a follow-up period of 52 weeks (9). Taking follow-up duration into account, we did not enroll in this trial as other trials had a relatively short duration time. However, SGLT-2 inhibitors did not show a better effect in lowering NT-proBNP levels in our meta-analysis. Trials with shorter-term or longer-term duration time are in need to make clear the effect on new-onset or chronic HFpEF.

Our finding shows the prospect of SGLT2i in the management of HFpEF. Previous studies and reviews of the SGLT-2 inhibitors in HF models illustrated some potential mechanisms of the cardioprotective effects which might help us understand how it works (41). First, SGLT-2 inhibitors show unique diuretic and natriuretic effects without neurohormonal activation or electrolyte disturbance (42, 43). This offers advantages in the management of volume status in patients with heart failure. Second, after treatment with SGLT2i, a metabolic switch occurs through reducing cardiac glucose oxidation and increasing utilization of ketone bodies and fatty acids (44, 45). Improvement of myocardial metabolism brings more efficient supply of energy and less accumulation of toxic products. An improved mitochondrial function may also be involved in the process (46). Third, LV diastolic function can be significantly ameliorated on account of reducing interstitial myocardial fibrosis and decreasing cardiomyocytes' stiffness (47). Besides, inhibition of myocardial Na+/H+ exchanger (NHE) and reduction of epicardial adipose can also make sense in the process (41). However, it is becoming more and more widely believed that rather than a simple glucose-lowering effect, the cardioprotective effect of SGLT2i is a combined pathophysiological process involving heart, kidney, vasculature, and even the whole body (48).

To sum up, treatment with SGLT-2 inhibitors makes a positive impact on prognosis outcomes in the HFpEF population and that may arise from the decrease of HHF. Although the incidence of CV death and all-cause death has not been proven to have a reduction, fewer events of rehospitalization indicate better quality of life and greater healthy status. Our finding of improvement in KCCQ-TSS conveys similar information. A higher score of KCCQ-TSS illustrated a present relief, at least symptomatically, after a short period of remedy of SGLT-2 inhibitors. What we mentioned above is consistent with the present therapeutic goal of reducing symptoms in HFpEF patients (3).

Based on the available results, SGLT-2 inhibitors could become a preferred choice in the following days when it comes to medications for HFpEF. In the 2022 AHA Guideline for the management of heart failure, the application of SGLT-2 inhibitors in HFpEF patients was first put forward and given a 2a class recommendation (49). As yet, there are still many ongoing or unpublished trials committing to clarifying the therapeutic effect of SGLT2i in the HFpEF population, and among them, the most compelling one may be the DELIVER trial, which is the largest and broadest trial in patients with HFmrEF or HFpEF intervening with SGLT2i (50). According to an internal announcement released by AstraZeneca, DELIVER confirmed that dapagliflozin reached a statistically significant and clinically meaningful reduction in the primary composite endpoint of CV death or worsening HF, and this is consistent with our conclusion. Complete results of DELIVER will be published in 2022 ESC Congress, and the efficacy of SGLT-2 inhibitors in HFpEF population will be further confirmed.

The limitations of this meta-analysis are as follows. First, the follow-up duration of included studies was diverse, from 12 weeks to 4.2 years, and that led to some selection when discussing certain outcomes to eliminate heterogeneity. Second, the LVEF cutoffs of included studies were variable. 2021 ESC Guideline and 2022 AHA Guideline all set LVEF ≥ 50% as a diagnostic criterion for HFpEF, but the number of studies completely met this standard was limited as LVEF > 40% or LVEF > 45% was thought general inclusion criteria in previous studies. Third, included statistics of reported outcomes were restricted because of the finite amount of original studies. Further subgroup analysis or regression analysis was not performed in this meta-analysis as well. Fourth, the majority of patients involved in our meta-analysis had diabetes. More evidence is needed to confirm the therapeutic effect in HFpEF patients without diabetes.

## Conclusion

As far as we know, our meta-analysis first illustrates remission of symptoms and improvement of prognosis at the same time in HFpEF patients using SGLT-2 inhibitors. Significant improvement in cardiovascular outcomes and health-related quality of life in HFpEF patients are explicated after the pooled estimate, and our results may provide support in therapeutic options and guideline development of HFpEF in the coming day.

## Data availability statement

The original contributions presented in the study are included in the article/Supplementary material, further inquiries can be directed to the corresponding author/s.

## Author contributions

DY: methodology, statistical analysis, quality assessment, and write and polish the draft. YZ: study selection, data collection, bias assessment, and write the draft. JY and ML: data visualization and consultation. FA: review and edit the manuscript. All authors reviewed the manuscript, gave their final approval, and agreed to be accountable for all aspects of the work ensuring integrity and accuracy.

## Funding

This study was supported by the National Natural Science Foundation of China (Nos. 81670325 and 81970368) and the Key Research and Development Project of Shandong Provence (No. 2019GSF108124).

## Conflict of interest

The authors declare that the research was conducted in the absence of any commercial or financial relationships that could be construed as a potential conflict of interest.

## Publisher's note

All claims expressed in this article are solely those of the authors and do not necessarily represent those of their affiliated organizations, or those of the publisher, the editors and the reviewers. Any product that may be evaluated in this article, or claim that may be made by its manufacturer, is not guaranteed or endorsed by the publisher.
