# Supplementary material for: SGLT-2 inhibitors on prognosis and health-related quality of life in patients with heart failure and preserved ejection fraction: A systematic review and meta-analysis
Source: Front Cardiovasc Med. 2022 Sep 8;9:942125. doi: 10.3389/fcvm.2022.942125 (PMC9492916; doi:10.3389/fcvm.2022.942125)
Supplement: Supplementary file 1 [file Data_Sheet_1.pdf]

Supplementary Materials

1 Searching Strategy

1) heart failure, diastolic
2) diastolic heart failures
3) diastolic heart failure
4) heart failure, preserved ejection fraction
5) heart failure with preserved ejection fraction
6) heart failure, normal ejection fraction
7) heart failure with normal ejection fraction
8) HFpEF
9) OR/1-8
10) heart failure
11) cardiac failure
12) heart decompensation
13) decompensation, heart
14) myocardial failure
15) congestive heart failure
16) heart failure, congestive
17) OR/10-16
18) sodium-glucose transporter 2 inhibitors
19) sodium glucose co-transporter type 2 inhibitor
20) sodium glucose cotransporter type 2 inhibitor
21) sodium glucose co-transporter 2 inhibitor
22) sodium glucose cotransporter 2 inhibitor
23) sodium dependent glucose co-transporter 2 inhibitor
24) sodium dependent glucose cotransporter 2 inhibitor
25) sodium dependent glucose transporter 2 inhibitor
26) sodium glucose linked cotransporter 2 inhibitor
27) sodium glucose linked transporter 2 inhibitor
28) SGLT-2 inhibitor
29) inhibitor, SGLT2
30) Gliflozins
31) Gliflozin
32) Dapagliflozin
33) Empagliflozin
34) Canagliflozin
35) Ipragliflozin
36) Luseogliflozin
37) Tofogliflozin
38) Ertugliflozin
39) Sotagliflozin
40) OR/18-39
41) randomized controlled trial

42) clinical trial, randomized
43) trial, randomized clinical
44) randomized controlled clinical trial
45) controlled clinical trial, randomized
46) OR/41-45
47) 9 AND 40 AND 46
48) 17 AND 40 AND 46
49) 47 OR 48

2 Supplementary Figures

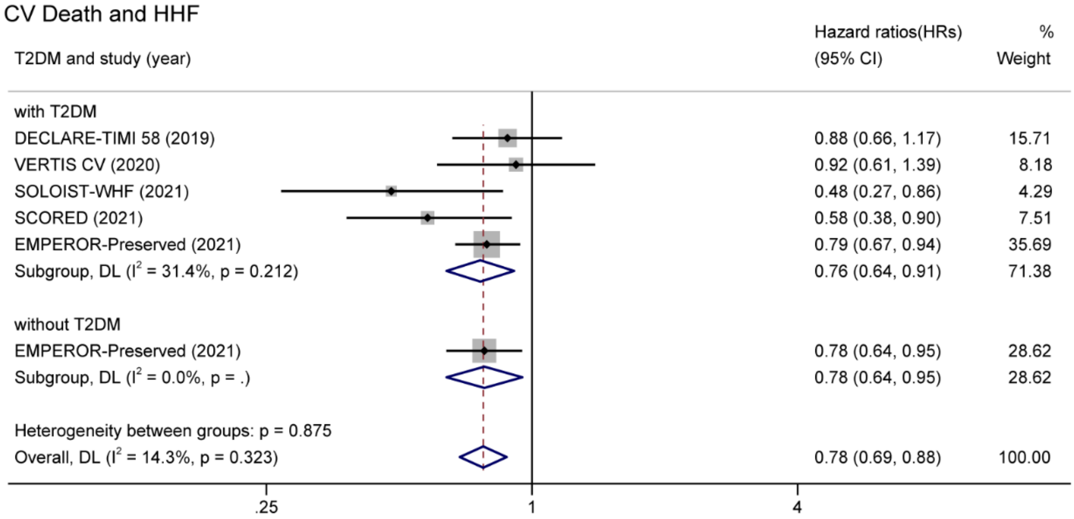

Supplementary Figure.1 Subgroup analysis of composite outcome of cardiovascular death and hospitalization for heart failure.

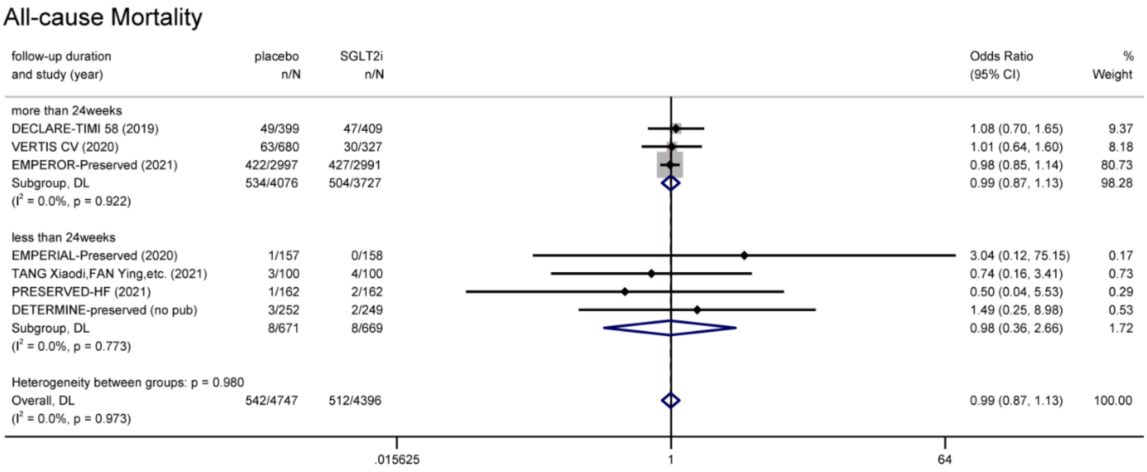

Supplementary Figure.2 Subgroup analysis of all-cause mortality.
